# Impact of Protein Coronas on Lipid Nanoparticle Uptake and Endocytic Pathways in Cells

**DOI:** 10.3390/molecules29204818

**Published:** 2024-10-11

**Authors:** Rui Wang, Jing He, Yuhong Xu, Baowei Peng

**Affiliations:** 1 College of Pharmacy, Dali University, No. 2 Hongsheng Road, Dali 671003, China; 13611528427@163.com (R.W.); m15969529279@163.com (J.H.); 2 Yunnan Key Laboratory of Screening and Research on Anti-Pathogenic Plant Resources from Western Yunnan, Dali University, Xueren Road, Dali 671003, China

**Keywords:** endocytosis, protein corona, LNP, phagosome

## Abstract

Lipid nanoparticles (LNPs), widely used in disease diagnosis and drug delivery, face the challenge of being surrounded by biological macromolecules such as proteins upon entering the human body. These molecules compete for binding sites on the nanoparticle surfaces, forming a protein corona. The impact of different types of protein coronas on LNP delivery remains unclear. In this study, we employed a newly developed, highly sensitive LNP labeling platform and analyzed the endocytosis of HeLa cells under different nutritional conditions using proteomics to address this critical issue. Our research found that under conditions of complete medium and amino acid starvation, most DNA-FITC vesicles in HeLa cells were located in the perinuclear region 4 h after transfection. In contrast, under serum starvation conditions, only a small portion of DNA-FITC vesicles were in the perinuclear region. On the other hand, through proteomics, we discovered that cells that were enriched in amino acids and complete medium contained more proteins, whereas those under serum starvation had relatively fewer enriched proteins. Through KEGG pathway enrichment analysis, we identified the phagosome and endocytosis pathways as particularly important. Lastly, differential analysis of proteins in these pathways revealed that proteins such as F-actin, Coronin, vATPase, TUBA, TUBB, MHCII, and TSP may have significant impacts on cellular endocytosis. Our research findings indicate that it is necessary to regulate cellular endocytosis based on different protein coronas to achieve optimal cytoplasmic release.

## 1. Introduction

In recent years, the development of nanobiomedical materials has experienced rapid and exponential growth. However, only a limited number of these materials have received approval for clinical applications. A significant disconnect exists between academia, industry, and research, primarily stemming from an incomplete understanding of the interactions between nanomaterials and biological systems. Upon introduction into the body, nanomaterials inevitably attract biological molecules, such as proteins, to their surface, forming what is known as a protein corona (PC) [1]. The PC serves as the initial biological barrier encountered in the biomedical applications of nanomaterials [2]. The composition of the protein corona is influenced by a variety of factors, including the inherent physicochemical properties of the nanomaterials, characteristics of the biological fluids, and environmental conditions [3,4]. This process of PC formation has the potential to modify the intrinsic physicochemical properties of the nanomaterials, leading to the acquisition of new chemical and biological features [5]. Therefore, achieving a deeper comprehension of the various types of PCs and their effects on the in vivo fate of nanomaterials is essential for establishing a robust scientific foundation to govern the efficacy and safety of nanomaterials.

Utilizing time-lapse microscopy imaging and software analysis allows for the differentiation and tracking of cells, extracting information such as their location and morphology [6]. Unlabeled live cell tracking generally employs phase contrast imaging, which can display the cell morphology more clearly. However, due to the low contrast between the cells and background in phase contrast images, analysis software finds it challenging to accurately identify and segment cells [7]. Consequently, many related cell tracking experiments still rely on manual tracking. High-content imaging systems combine microscopic imaging with multi-parameter quantitative image analysis technologies, enabling the objective collection and analysis of multiple parameters at the single-cell level. They are widely used in areas such as cell viability, cell cycle, and toxicity testing [8,9]. The digital phase contrast (DPC) module of these systems constructs digital phase contrast images from brightfield images, significantly improving the signal-to-noise ratio. This allows for the automatic and accurate identification and tracking of fluorescently labeled cells. The system can analyze various data results, such as the number of cells being tracked in real-time, their area, changes in the fluorescence signal intensity, texture parameters, and cell dynamics characteristics, thus reducing the experimental complexity and cost.

Through the use of a highly sensitive LNP tracking platform and proteomic analysis, this study reveals how nutrient-regulated protein coronas affect the endocytic activity of LNPs. Our research not only provides a systematic comparison of the effects of nutritional levels on endocytic activities but also proposes potential mechanistic explanations.

## 2. Results

### 2.1. Characterization of Intracellular Uptake Activity of DNA-LNPs at Different Time Points

We plan to investigate the endocytosis of LNP-DNA at different time points and verify the imaging tracking capabilities of our high-content system. Streptavidin (SA) has an extremely high affinity for biotin, with one SA molecule being capable of binding to four biotin molecules in an irreversible manner. SA-FITC is a fluorescent probe that combines the fluorescent dye FITC with streptavidin to detect biotinylated antibodies, proteins, nucleic acids, or other molecules [10]. GFP-Rab7 plasmid was used as a template to obtain linear DNA by PCR amplification with 15% of dUTP-11-biotin. The dUTP-11-Biotin DNA-LNPs obtained as described above were transfected into cells in Complete Medium Fed (500 mL glucose DMEM + 10% fetal bovine serum + 1% streptomycin) for 2, 4, 8, and 24 h; the cells were then fixed, stained, and imaged using high-content imaging systems.

**Figure 1:**
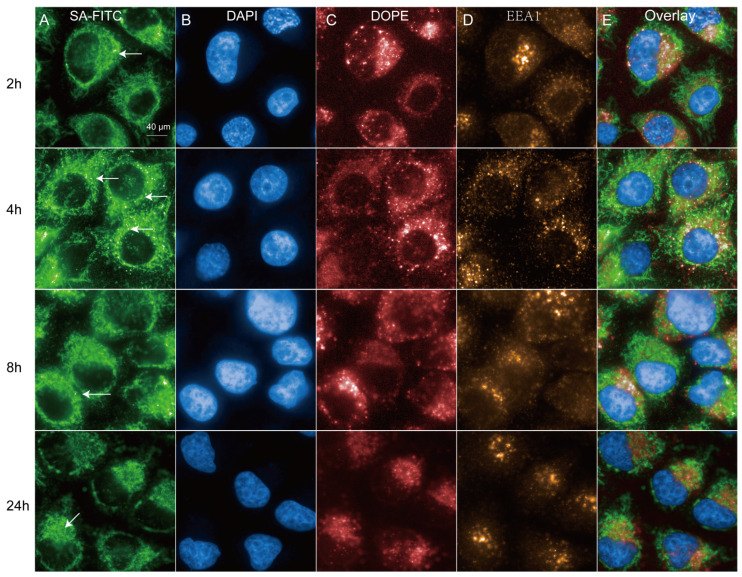
Characterization of intracellular uptake of dUTP-11-Biotin LNPs at different time points. (**A**) Endocytosis of LNPs DNA-FITC in HeLa cells pulsed with 2.5 µg DNA-FITC encapsulated in LNPs for 2 h, 4 h, 8 h, and 24 h. (**B**) HeLa cells were labeled with DAPI. (**C**) LNPs/lipids were labeled with DOPE-atto647. (**D**) LNPs/lipids were labeled with EEA1. Arrowheads point to LNP-DNA in perinuclear “cloud”, and arrows point to individual cytoplasmic endosomes. (**E**) Representative merged images (Overlay) at indicated time points.

After 4 h of transfection, most of the DNA-FITC vesicles in the fed cells were located in the perinuclear region (Figure 1A,B), whereas fewer DNA-FITC vesicles were observed in the perinuclear region at 2, 8, and 24 h. Furthermore, we found that most DNA particles did not significantly colocalize with the early endosome marker EEA1 (Figure 1D), suggesting that these DNA particles are not taken up by the cells through the classical endocytosis pathway. Similarly, DOPE-Atto647 (0.1% molar ratio) was included in the formulation to track the lipids of the LNP. In fed cells pulsed with LNP for 4 h, numerous small peripheral LNP-endosomes were observed, and most of them also did not colocalize with endosomes (Figure 1C). In conclusion, these results validate the sensitivity of our system and indicate that the formation of peripheral endosomes following LNP-DNA uptake peaks at 4 h.

### 2.2. Characterization of Endocytic Activity in Cells Undergoing Nutrient Depletion

**Figure 2:**
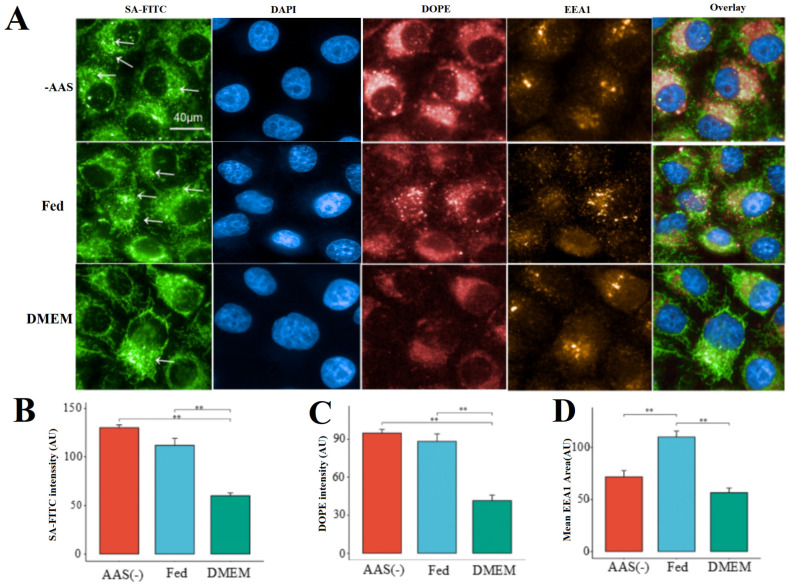
The internalization of LNPs varies depending on different nutrients. (**A**) Correlation of peripheral LNP endosomes with endocytosis activity in AAs(−) HeLa cells, Fed HeLa cells, and DMEM HeLa cells. (Arrows point to individual cytoplasmic endosomes.) (**B**) Quantification of SA-FITC intensity. (**C**) Quantification of DOPE intensity. (**D**) Quantification of EEA1 area. ** *p* < 0.01.

After validating our platform, we began to investigate the relationship between the internalization of LNPs and cellular nutrient availability. We hypothesized that during the robust endocytic activity of cells, the formation of peripheral LNP endosomes relies on changes in PC induced by variations in the nutrient availability. We transfected dUTP-11-biotinylated DNA-LNPs into cells that were deprived of specific nutrients (especially amino acids, AAs), fed cells, and cells lacking serum (DMEM) for 4 h, followed by cell fixation, staining, and imaging using a high-content imaging system. We investigated the presence of small peripheral blood LNP nuclear endosomes in HeLa cells cultured in DMEM, -AAs, and Fed conditions. Compared to DMEM cells, a large number of LNP nuclear endosomes were observed in the perinuclear region of both the Fed and -AAs cells (Figure 2A,B). Furthermore, in the Fed and AAS cells, LNPs (DOPE-Atto647) accumulated around the nucleus (Figure 2A). In the DMEM cells, there was less accumulation of LNPs (DOPE-Atto647) around the nucleus, possibly due to the rapid degradation of LNPs (DOPE-Atto647) in DMEM cells lacking fetal bovine serum (Figure 2A,C). In the Fed cells, a large number of EEA1+ early endosomes were found around the nucleus (Figure 2A,D), whereas in the -AAs and DMEM cells, there were fewer EEA1+ early endosomes around the nucleus (Figure 2A,C). We hypothesize that after LNP transfection, rapid binding with fetal bovine serum to form a PC, mediated by certain amino acids, may lead to the aggregation of EEA1+ early endosomes around the nucleus.

### 2.3. LNP-PC-Based Proteomic Analysis Strategy

**Figure 3:**
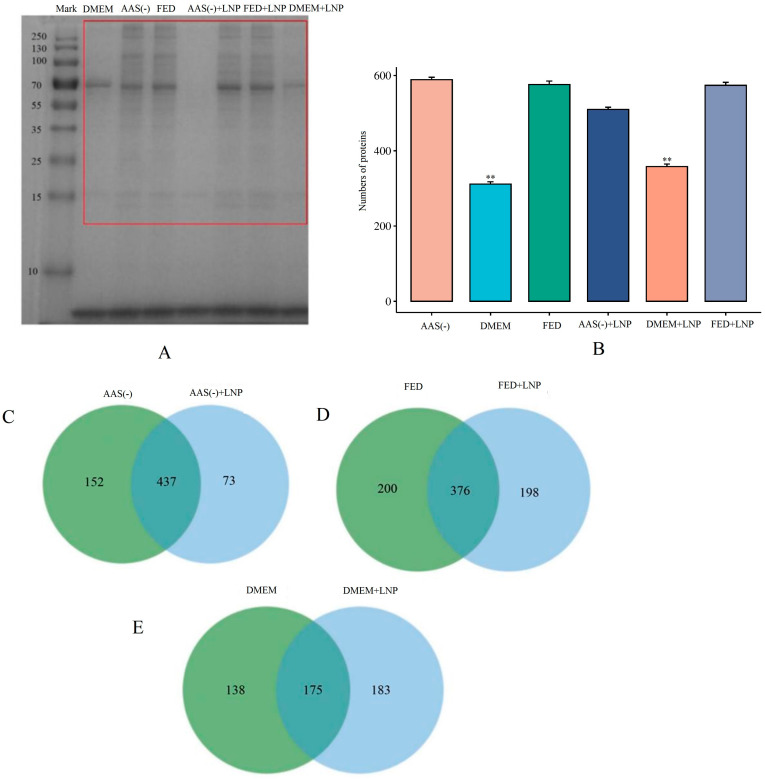
Protein corona analysis of LNPs. (**A**) SDS-PAGE gel indicating that the compositions of corona proteins DMEM, AAS(-), FED, AAS(-), AAS(-) + LNP, FED + LNP, and DMEM + LNP were co-incubated with serum for 8 h. (The bands within the red box are the protein bands sent for examination.) (**B**) The number of proteins. (**C**) A Venn diagram illustrating the unique and shared proteins between AAS(-) and AAS(-) + LNP. (**D**) A Venn diagram illustrating the unique and shared proteins between FED and AAS(-) + LNP. (**E**) A Venn diagram illustrating the unique and shared proteins between DMEM and DMEM + LNP (** *p* < 0.01).

A PC is considered a determining factor for the overall biological behavior and therapeutic efficacy of liposomes [11]. We speculated that the significant differences in cellular endocytic capabilities described above may be attributed to changes in the PC. To understand the underlying mechanisms of the PC, we collected the supernatant from cells transfected with LNPs-PC for 4 h in AAS, Fed, and DMEM cells, added streptavidin magnetic beads, and allowed them to incubate for 2 h. Finally, the samples were analyzed by SDS-PAGE gel electrophoresis. As shown in Figure 3A (see Figure S1 for the raw gel plots), the protein corona composition of the DMEM group exhibited significant differences compared to the AAS(-) and FED groups, suggesting that the DMEM group altered the surface properties of the lipid nanoparticles (LNPs), thereby affecting the types of proteins that were adsorbed on the LNPs. Additionally, to reduce interference from other impurities, we excised the protein bands within the red box in Figure 3A for LC-MS sequencing (each sample was repeated three times). We performed PC identification using the Uniprot-Homo sapiens (version 2023, 20,610 entries) database. Shown in Figure 3B, 589, 313, 576, 510, 358, and 574 kinds of proteins were identified in the PCs of AAS(-), DMEM, AAS(-) + LNP, DMEM + LNP, and FED + LNP (Supplementary Data S1). In addition, there were significant differences between the AAS and FED groups compared to the DMEM group (*p* < 0.01). Similarly, the AAS + LNP and FED + LNP groups also showed significant differences compared to the DMEM + LNP group (*p* < 0.01). Next, we performed a Venn diagram analysis on the above six groups. Figure 3C–E show that there are 437 unique and shared proteins between AAS(-) and AAS(-) + LNP, 376 unique and shared PC counts between FED and FED + LNP, and 176 unique and shared PC counts between DMEM and DMEM + LNP. We found that both the individually detected and shared PC counts in the DMEM group were significantly fewer than those in the AAS(-) and FED groups. Albumin, IgG, IgM, Complement factor C3, and ApoJ (clusterin) were not detected in our samples. These proteins are high-abundance proteins in the plasma, which are easily masked in proteomics, making it difficult to detect low-abundance proteins. Therefore, when the protein corona data are further used to evaluate the impact on cells, cells are typically supplemented with fetal bovine serum (FBS: characterized by lower protein concentrations than human serum, particularly in terms of typical conditioning proteins, immunoglobulins, and complement proteins) [12,13,14]. Therefore, we chose to create a protein corona in fetal bovine serum. We speculate that these moderate-to-low-abundance plasma proteins may affect the cellular endocytic capacity.

### 2.4. KEGG Enrichment Analysis

**Figure 4:**
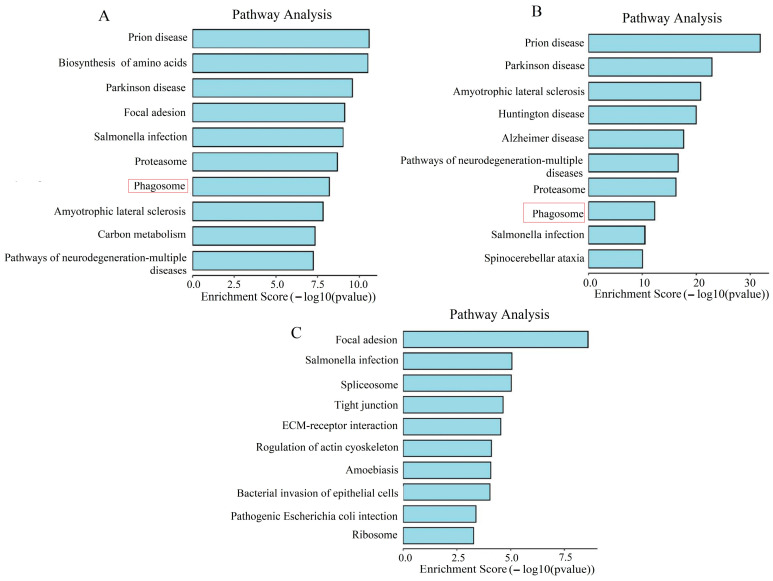
KEGG enrichment analysis; (**A**) KEGG analysis of the AAS(-) group; (**B**) KEGG analysis of the FED group; (**C**) KEGG analysis of the DMEM group (The red box contains the same signaling pathway).

KEGG (Kyoto Encyclopedia of Genes and Genomes) enrichment analysis is a bioinformatics method that involves comparing a list of genes from a study with information on metabolic pathways, signaling pathways, and other biological processes available in the KEGG database to identify which KEGG pathways show statistically significant enrichment of genes [15]. This analysis helps us understand which biological processes or functional pathways are important in our study, revealing potential mechanisms of endocytosis or signaling pathways. We selected “Homo sapiens” as the species, with a screening criterion of *p*-value < 0.05. In the AAS(-) group, we enriched a total of 60 pathways (Supplementary Data S2). In Figure 4A, the top 10 enriched pathways include pathways of neurodegeneration—multiple diseases, Carbon metabolism, Amyotrophic lateral sclerosis, phagosome, Proteasome, Salmonella infection, Focal adhesion, Parkinson disease, Biosynthesis of amino acids, and Prion disease. A phagosome is a cytoplasmic vesicle within a cell that contains solid particles or microorganisms ingested by the cell. In this pathway, cells engulf solid particles (such as bacteria, viruses, cell debris, etc.) to eliminate foreign or infectious agents. The phagosome pathway involves a series of complex intracellular events, including the uptake of particles, vesicle formation, intracellular transport, fusion, and the digestion and degradation of contents within the vesicle. In the phagosome pathway, cells engulf solid particles to form phagosome vesicles, which then fuse with lysosomes, where the contents within the particles are ultimately degraded and digested. This process plays a critical role in cells engulfing pathogens, cell debris, and other harmful substances, maintaining immune homeostasis in the body and protecting against external harmful substances. Therefore, we conclude that phagosome is important for cellular endocytosis.

In the FED group, we enriched a total of 186 pathways (Supplementary Data S3). In Figure 4B, the top 10 enriched pathways include Spinocerebellar ataxia, Salmonella infection, Phagosome Proteasome, pathways of neurodegeneration—multiple diseases, Alzheimer disease, Huntington disease, Amyotrophic lateral sclerosis, Parkinson disease, and Prion disease. Among them, phagosome is also included in the top 10 signaling pathways.

In the DMEM group, we enriched a total of 186 pathways (Supplementary Data S4). In Figure 4C, the top 10 enriched pathways include Ribosome, Pathogenic Escherichia coli infection, bacterial invasion of epithelial cells, Amoebiasis, regulation of actin cytoskeleton, ECM—receptor interaction, Tight junction, Spliceosome, Salmonella infection, and Focal adhesion.

### 2.5. Phagosome and Differential Analysis

**Figure 5:**
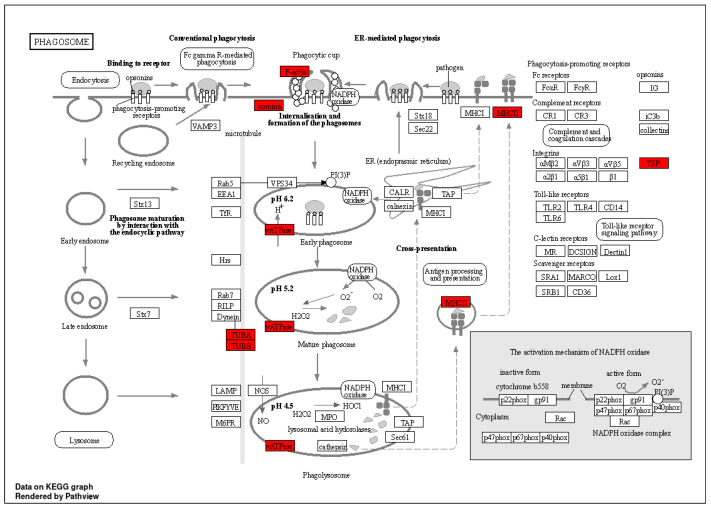
The signaling pathway diagram of phagosome (the images are sourced from the DAVID database 2024.05).

**Figure 6:**
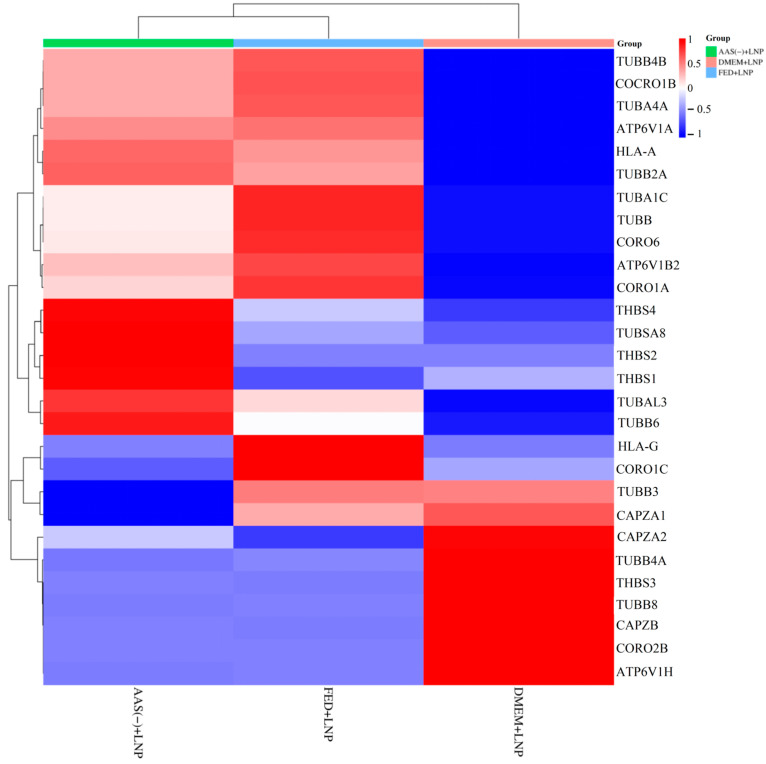
Cluster heatmap analysis.

Figure 5 shows the complete signal pathway of the phagosome in KEGG, in which the proteins enriched by proteomics include Filamentous actin (F-actin), Coronin, Vacuolar-ATPase (vATPase), Tubulin, Alpha (TUBA), Tubulin, Beta (TUBB), Major Histocompatibility Complex class II (MHCII), and Thrombospondin (TSP), highlighted in red. We performed gene name conversion for the proteins mentioned above, as shown in Table 1. Based on the measured peak areas, we conducted a cluster heat map analysis, as shown in Figure 6. We observed that the AAS(-) + LNP group and the FED + LNP group clustered closely together, while the DMEM + LNP group formed a separate column. The DMEM + LNP group exhibited relatively weaker phagocytic capability. Figure 6 indicates that genes such as TUBB4B, Coronin-1B, TUBA4A, ATP6V1A, HLA-A, TUBB2A, TUBA1C, TUBB, CORO6, ATP6V1B2, CORO1A, THBS4, TUBS1, TUBAL3, TUBB6, HLA-G, and CORO1C, among others, were significantly downregulated compared to the AAS(-) + LNP and FED + LNP groups. Conversely, genes such as TUBB3, CAPZA1, CAPZ2, TUBB4A, THBS3, TUBB8, CAPZB, and CORO2B were markedly upregulated. Therefore, we speculate that the expression of these genes ultimately affected the phagocytic capability of the DMEM + LNP group.

## 3. Discussion

In this study, in order to understand the escape of endocytic vesicles in a spatiotemporal context, we utilized a highly sensitive LNP tracking platform and combined it with the definition of cellular endocytic activity states to systematically study whether different types of PCs affect intracellular transport. We believe that proteins such as F-actin, Coronin, vATPase, TUBA, TUBB, MHCII, and TSP are ultimately going to affect the ability of cells to endocytose. F-actin is a fundamental component of the cytoskeleton that participates in cell morphology, migration, and intracellular transport. Studies show that under nutrient-rich conditions, the polymerization of actin can be enhanced, promoting cell migration and processes such as endocytosis. Coronin is a protein that binds to actin and regulates actin dynamics, participating in cell migration and phagocytosis. In conditions of nutrient deprivation, the activity of Coronin may decrease, affecting endocytosis and autophagy processes [16]. vATPase is crucial for the acidification of intracellular compartments, which is essential for protein degradation, receptor recycling, and other cellular processes. Under nutrient-rich conditions, vATPase activity supports these processes, facilitating rapid turnover and recycling [17]. Similarly, the polymerization of microtubule proteins, such as TUBA and TUBB, increases to support cell division and intracellular transport. The nutritional status can significantly affect microtubule dynamics; during nutrient deprivation, modifications such as acetylation and deacetylation of microtubule proteins may occur, altering the microtubule’s stability and function to adapt to stress [18]. MHCII molecules are involved in antigen presentation and immune responses. Nutrient-rich conditions can enhance an MHCII-mediated antigen presentation by promoting protein synthesis and transport [19]. TSP is a matrix protein that participates in cell adhesion, migration, and tissue remodeling. The nutritional status can affect the levels and activity of TSP. In nutrient-rich conditions, TSP expression can be upregulated, promoting interactions between cells and remodeling of the extracellular matrix. Conversely, nutrient deprivation may reduce TSP expression, impacting processes such as wound healing and angiogenesis [20]. In addition, Apo A1 is one of the most abundant proteins in the protein corona, isolated by centrifugation after incubation with FBS; however, it has no effect on the cellular uptake of LNPs [21]. On the other hand, Chen et al. [22] concluded that Apo E, present in protein coronas of nearly neutral PEGylated and non-PEGylated LNPs, was involved in the facilitation of the LNP uptake by the HepG2 cells in the presence of FBS.

Previously, it has been proposed that the uptake of LNPs depends on receptor-mediated endocytosis, and the cellular endocytic activity towards LNPs is closely related to lysosomal activity [23]. We found that the endocytic capacity of LNPs is minimal in DMEM (Figure 2). On the other hand, in cells deprived of AAs(-) and in FED, the endocytic activity of LNPs significantly increases. Through proteomic sequencing, we identified that ATP6V1A and ATP6V1B2 are significantly downregulated in the DMEM group (Figure 6). ATP can provide energy to maintain the activity of enzymes and membrane proteins inside lysosomes, ensuring the normal digestion of waste materials. Therefore, we speculate that ATP affects lysosomal activity, resulting in decreased endocytic capacity in the DMEM. Studies have shown that the presence of PC can be directly observed by using transmission electron microscopy (TEM) and scanning electron microscopy (SEM) to visualize PCs adsorbed on the surface of nanoparticles, thus confirming the presence of PCs [24]. However, this method does not allow for quantitative analysis. To further investigate the protein corona composition, proteins are typically separated and desorbed from purified protein–nanoparticle complexes. We utilized the sodium dodecyl sulfate polyacrylamide gel electrophoresis (SDS-PAGE) method by cutting protein gel bands and then performing protein sequencing using LC-MS/MS, which allows for both qualitative and quantitative analysis of PCs (Figure 3). The current mechanisms underlying how the protein corona affects cell metabolism are not yet clear. It is known that proteins in the corona on nanoparticles can interact with other biomolecules inside and outside the cells. The intracellular protein corona regulates the macrophage phenotype and induces stress responses in endothelial cells. We believe that intercellular proteins replace some proteins from the surface of nanoparticles, disrupting protein homeostasis, subsequently triggering the “Neutrophil activation involved in immune response”, “Respiratory vesicle lumen”, “Cytoplasmic vesicle lumen”, and “Secretory granule lumen” pathways (Figure 4). The PC-induced neutrophils impact the cellular clearance pathways of nanoparticles and alter cell metabolism. Currently, the targeted delivery of nanomedicines still presents significant challenges. Research by Wilhelm et al. has shown that only 0.7% of nanomaterials can be effectively transported to specific target sites [25]. Ideally, drugs should be enriched in target organs while minimizing accumulation in other tissues. However, in most cases, the formation of the protein corona weakens the targeting ability of nanomaterials. This is because the PC covers the surface of the nanomaterial carrying ligands, creating a barrier between the ligands and target cells, impeding their mutual recognition and reducing targeting efficiency. Therefore, in the follow-up experiments, we will knock out or inhibit proteins such as F-actin, Coronin, vATPase, TUBA, TUBB, MHCII, and TSP to elucidate the impact of these protein coronas on the mechanism of cellular phagocytosis.

## 4. Conclusions

By employing a highly sensitive LNP tracking platform combined with cell-specific endocytic activity states, we discovered that different types of protein coronas influence the endocytic pathways of LNPs within cells (such as F-actin, Coronin, vATPase, etc.). Therefore, regulating cellular endocytosis based on different protein coronas is crucial for achieving optimal cytoplasmic release.

## 5. Materials and Methods

### 5.1. Antibodies and Reagents

Rabbit anti-EEA was obtained from Cell signaling technology (CST, Boston, MA, USA). Goat anti-rabbit and goat anti-mouse secondary antibodies, labeled with Alexa Fluor 555, were obtained from Molecular Probes (Silicon Valley, CA, USA). DOPE-Atto 647 was obtained from Atto-Tec (Berlin, Germany). Streptavidin-FITC was obtained from APE*BIO (Houston, TX, USA). dUTP-11-biotin was obtained from Thermo Fisher (Waltham, MA, USA). Phanta Max Super-Fidelity DNA Polymerase was obtained from Vazyme (Nanjing, China). The dNTP solution set was obtained from US Everbright (Silicon Valley, CA, USA). Cholesterol was obtained from Sigma-Aldrich (St. Louis, MO, USA). SM102, DSPC, and DMG-PEG were obtained from Sinopeg (Xiamen, China). Coomassie Brilliant Blue (CBB) and streptavidin magnetic beads were obtained from Beyotime (Shanghai, China).

### 5.2. Linear DNA Amplification

GFP-Rab7 plasmid was used as a template to obtain the linear DNA by PCR amplification with 15% of dUTP-11-biotin. The forward primer was 5′-gaccccggcgccgccaccatggtgagcaagggcgaggagctgttc. The reverse primer was 5′-ccaaggcacacgtggtccatcgtgtacagctcgtccatgccga. The PCR product was purified by ammonium acetate precipitation of DNA.

### 5.3. Nucleic Acid Formulation into Lipid Nanoparticles

LNPs were prepared with the ionizable lipid SM102, distearoylphosphatidylcholine (DSPC), cholesterol, and PEG–DMG. A DOPE-Atto 647-labeled lipid was added to the ethanol phase at a content of 0.1% of the mass fraction of the four lipids. An aqueous phase (nucleic acids (linear DNA) diluted in 20 mM citrate buffer, pH 4) and organic phase (lipids dissolved in 90% ethanol solution) were mixed at a 3:1 ratio through syringe mixing or using a microfluidic mixer (Microfluidics, Washington, WA, USA). The ethanol was then removed, and the external buffer was replaced with water (NaCl, 20 mM Tris pH 7.4). Benchmark LNPs had a component molar ratio of 50:10:38.5:1.5 (ionizable lipid/disteroylphosphatidyl choline/cholesterol/PEG-DMG) and N/P ratio of 6. Particle size and zeta were determined using a Malvern Zetasizer advance Pro (Malvern, UK). The DNA LNP content was determined by agarose gel electrophoresis with or without treating an aliquot of the LNP solution with an equal volume of Triton-X 100 solution (10% in water) and then vortexing and incubating it at 37 °C for 5 min.

### 5.4. Stains

To track LNPs, streptavidin-FITC was used to track the cargo DNA containing 11-biotin- dUTP. Cell nuclei were counterstained with 4′,6-diamidino-2-phenylindole (DAPI) for 10 min at 1 µg/ mL.

### 5.5. Cell Culture

HeLa cells were originally purchased from ATCC (CRM-CCL-2) (Mansas, VA, USA). Cultures were maintained under standard conditions (37 °C, 5% CO_2_, 95% humidity) in glutamine-containing Dulbecco’s Modified Eagle’s Medium (DMEM) (#11965118, Gibco, Grand Island, NY, USA), supplemented with 10% fetal bovine serum (FBS) (#A0500-3010, Cegrogen) (Eupen,Belgium) and 1% penicillin-streptomycin (#15250062, Thermo Fisher, Waltham, MA, USA). For transfection and imaging, cells were plated on 24-well plates at 8 × 10^4^/well in slides treated with Poly-L-lysine and then returned to the incubator to adhere and acclimatize overnight. If necessary, cells were deprived of nutrients for 4 h prior to incubation with LNPs. For serum deprivation (-FBS), cells were cultured in a medium containing plain DMEM. For AA deprivation (-AAs), cells were cultured in a medium containing customized DMEM without AAs and 10% FBS dialyzed against PBS (pH 7.2, 10 mM) by MWCO 3500 overnight to remove the AAs. Then, LNPs (containing 2.5 µg with linear DNA) were added to cells in 500 µL medium, followed by transfection for 4 h.

### 5.6. Immunofluorescence and Uptake Assay

Cells were grown on coverslips and pulsed with DNA-dUTP-11-biotin-loaded LNPs for various times. For in vitro immunofluorescence analysis, cells were fixed in 4% paraformaldehyde at room temperature for 30 min, followed by two washes with PBS. Subsequently, the cells were permeabilized with 100 µg/mL digitonin for 20 min and blocked in a 1.5% BSA solution. The cells were then incubated overnight at 4 °C with rabbit anti-EEA in 1.5% BSA and intensively washed 3 times for 5 min with PBST. Alexa Fluor 555 and streptavidin-FITC, complemented with DAPI, were applied for 4 h at room temperature. After intensive washing, coverslips were mounted on glass-slides. Images were acquired with Operetta CLS high-content analysis system (2.0) with 63× Water Immersion Objective (NA 1.15). Multiple layers of images along the z-axis were acquired. The images were analyzed with Harmony software (Harmony 22) or ImageJ (ImageJ 2.0).

### 5.7. Coomassie Brilliant Blue

We collected the supernatant containing LNPs (with 2.5 µg of linear DNA) from the previous step (transfection for 4 h) and added it to the cells in a 1.5 mL centrifuge tube. We combined it with 30 µL of streptavidin magnetic beads, incubated it for 2 h, and then discard the supernatant. The beads were then lysed at 100 °C for 10 min in 60 µL of lysis buffer (1% Triton X-100, 20 mM Hepes, 150 mM sodium chloride, 12.5 mM disodium β-glycerophosphate, 1.5 mM magnesium chloride, 2 mM EGTA). We collected the supernatant as the sample, loaded the samples onto a 10% SDS-PAGE gel, and ran it at 80 V for 1 h, followed by 120 V until the bromophenol blue reached around 1.5 cm above the stacking gel. We stained the protein bands with Coomassie Brilliant Blue for 30 min, destained them overnight with ultrapure water, and visualized it on an imager.

### 5.8. LC-MS/MS Analysis

After washing with ultrapure water, the SDS-PAGE gel was cut into small pieces of 1 mm squares and put in an Eppendorf tube (Servicebio, Wuhan, China). Then, 500 μL of destaining solution, which consisted of 50 mM ammonium bicarbonate(NH_4_HCO_3_) and 50% acetonitrile (ACN), was used for decolorizing until the gel pieces became colorless at 37 °C, 600 rpm. After washing with 50 mM ammonium bicarbonate(NH_4_HCO_3_), the gel pieces were solidified with acetonitrile (ACN). Then, the gel pieces were reduced with 10 mM dithiothreitol (DTT), which was dissolved with 50 mM ammonium bicarbonate (NH_4_HCO_3_) at 37 °C, 500 rpm, for 1 h. After cooling to room temperature, acetonitrile (ACN) was added to solidify the gel. Then, 55 mM iodoacetamide (IAM), which was dissolved with 50 mM ammonium bicarbonate(NH_4_HCO_3_), was added to the gel to alkylate the sample at room temperature for 40 min in darkness. After washing with 50 mM ammonium bicarbonate(NH_4_HCO_3_), the gel pieces were solidified with acetonitrile (ACN). An appropriate volume of 13 ng/μL trypsin solution, which was dissolved with 50 mM ammonium bicarbonate(NH_4_HCO_3_), was added to digest the gel pieces and incubated at 37 °C overnight. Trifluoroacetic acid (TFA) was added to the sample to a final concentration of 1% to terminate the digestion process. Finally, 0.1% trifluoroacetic acid (TFA) with 60% acetonitrile (TFA) and 0.1% trifluoroacetic acid (TFA) with 90% acetonitrile (TFA) was used to extract 30 peptides from the gel pieces. The peptide was desalted by a Pierce C18 Spin Tips and dried in a speed vacuum concentrator.

The peptides were re-dissolved in solvent A (A: 0.1% formic acid in water) and analyzed using an Orbitrap Exploris 480 coupled to an EASY-nanoLC 1200 system (Thermo Fisher Scientific, Waltham, MA, USA). Then, 2 μL peptide sample was loaded onto a 25 cm analytical column (75 μm inner diameter, 1.9 μm resin (Dr Maisch) (Ammerbuch, Germany) and separated with 60 min-gradient starting at 2.2% buffer B (80% ACN with 0.1% FA) followed by a stepwise increase to 90% in 54.5 min, 99% in 0.5 min, and remaining there for 5 min. The column flow rate was maintained at 350 nL/min, with a column temperature of 40 °C. The electrospray voltage was set to 2 kV. The mass spectrometer (Thermo Fisher, Waltham, MA, USA) was run under data-dependent acquisition (DDA) mode and automatically switched between MS and MS/MS mode. The survey of the full scan MS spectra (m/z: 350–1500) was acquired in the Orbitrap (Thermo Fisher, Waltham, MA, USA) with a 60,000 resolution. A Normalized automatic gain control (AGC) target of 300% and a maximum injection time of 25 ms were used. Then, the precursor ions were selected into the collision cell for fragmentation by higher-energy collision dissociation (HCD), and the normalized collection energy was 30%. The MS/MS resolution was set at 15,000, the Normalized automatic gain control (AGC) target was 50%, the maximum injection time was 22 ms, and the dynamic exclusion was 30 s.

### 5.9. Data Processing

Tandem mass spectra were processed by PEAKS Studio version 10.6 (Bioinformatics Solutions Inc., Waterloo, ON, Canada). The database was Homo_sapiens (version 2023, 20,610 entries), which we downloaded from uniprot. Trypsin was set as the digestion enzyme, and Semi-specific was specified as the digestion type. PEAKS DB was searched with a fragment ion mass tolerance of 0.02 Da and a parent ion tolerance of 10 ppm. The max missed cleavage was 2. Carbamidomethyl on Cysteine was specified as the fixed modification. Oxidation on methionine, Deamidation on asparagine and glutamine, and Acetylation on Protein N-term were specified as the variable modifications. The peptides with 1% FDR and the proteins with 1% FDR and containing at least 1 unique peptide were filtered.

### 5.10. Statistical Analyses

The densitometry of immunoblots and intensity of fluorescence signals were quantified with the imageJ software. Data are expressed as mean ± standard deviation (SD). Statistical significance was calculated by one-way analysis of variance (ANOVA) with a Tukey 31 post hoc test for multiple comparisons or two-tailed Student’s *t*-test for two-group comparisons. *p* ≤ 0.05 was considered statistically significant.

## Figures and Tables

**Table 1:** Protein and gene name conversion.

Name	Protein Description	Gene Name
F-actin	F-actin-capping protein subunit alpha-2	CAPZA2
	F-actin-capping protein subunit alpha-1	CAPZA1
	F-actin-capping protein subunit beta	CAPZB
Coronin	Coronin-1A	CORO1A
	Coronin-1B	CORO1B
	CORO-2B	CORO2B
	Coronin-6	CORO6
	Coronin-1C	CORO1C
vATPase	V-type proton ATPase catalytic subunit A	ATP6V1A
	V-type proton ATPase subunit B, brain isoform	ATP6V1B2
	V-type proton ATPase subunit H	ATP6V1H
TUBA	Tubulin alpha-4A chain	TUBA4A
	Tubulin alpha-1C chain	TUBA1C
	Tubulin alpha-8 chain	TUBA8
	Tubulin alpha chain-like 3	TUBAL3
TUBB	Tubulin beta chain	TUBB
	Tubulin beta-4B chain	TUBB4B
	Tubulin beta-2A chain	TUBB2A
	Tubulin beta-4A chain	TUBB4A
	Tubulin beta-3 chain	TUBB3
	Tubulin beta-6 chain	TUBB6
	Tubulin beta-8 chain	TUBB8
MHCII	HLA class I histocompatibility antigen, alpha chain G	HLA-G
	HLA class I histocompatibility antigen, A alpha chain	HLA-A
TSP	Thrombospondin-1	THBS1
	Thrombospondin-2	THBS2
	Thrombospondin-3	THBS3
	Thrombospondin-4	THBS4

## Data Availability

The data that support the findings of this study are available from the corresponding author upon reasonable request.

## Supplementary Materials

The following supporting information can be downloaded at https://www.mdpi.com/article/10.3390/molecules29204818/s1: Figure S1: Kaomasi Bright Blue Original Adhesive Diagram; Supplementary Data S1: Number of protein crown identification types; Supplementary Data S2: The signaling pathway of AAS (-); Supplementary Data S3: The signaling pathway of Fed; Supplementary Data S4: The signaling pathway of DMEM.

## References

[1] Vroman L. (1962). Effect of Adsorbed Proteins on the Wettability of Hydrophilic and Hydrophobic Solids. Nature.

[2] Nienhaus K., Nienhaus G.U. (2023). Mechanistic Understanding of Protein Corona Formation around Nanoparticles: Old Puzzles and New Insights. Small.

[3] Abid N., Khan A., Shujait S., Chaudhary K., Ikram M., Imran M., Haider J., Khan M., Khan Q., Maqbool M. (2022). Synthesis of nanomaterials using various top-down and bottom-up approaches, influencing factors, advantages, and disadvantages: A review. Adv. Colloid Interface Sci..

[4] Lee H. (2023). Differences in protein distribution, conformation, and dynamics in hard and soft coronas: Dependence on protein and particle electrostatics. Phys. Chem. Chem. Phys..

[5] Bashiri G., Padilla M., Swingle K., Shepherd S., Mitchell M., Wang K. (2023). Nanoparticle protein corona: From structure and function to therapeutic targeting. Lab A Chip.

[6] Piltti K.M., Cummings B.J., Carta K., Manughian-Peter A., Worne C.L., Singh K., Anderson A.J. (2018). Live-cell time-lapse imaging and single-cell tracking of in vitro cultured neural stem cells Tools for analyzing dynamics of cell cycle, migration, and lineage selection. Methods.

[7] Jaccard N., Szita N., Griffin L.D. (2017). Segmentation of phase contrast microscopy images based on multi-scale local Basic Image Features histograms. Comput. Methods Biomech. Biomed. Eng. Imaging Vis..

[8] Booij T.H., Price L.S., Danen E.H.J. (2019). 3D Cell -Based Assays for Drug Screens: Challenges in Imaging, Image Analysis, and High-Content Analysis. SLAS Discov..

[9] Gunn A.L., Yashchenko A.I., Dubrulle J.J., Hatch Emily M. (2024). A high-content screen reveals new regulators of nuclear membrane stability. Sci. Rep..

[10] Chen Cherry C., Borden Mark A. (2010). Ligand conjugation to bimodal poly(ethylene glycol) brush layers on microbubbles. Langmuir.

[11] Wienke H.S., Pinilla B.M.C., Contri V.R., Brandelli A. (2024). Development of Polyelectrolyte-Coated Liposomes as Nanostructured Systems for Nisin Delivery: Antimicrobial Activity and Long-Term Stability. Food Biophys..

[12] Palchetti S., Colapicchioni V., Digiacomo L., Caracciolo G., Pozzi D., Capriotti A.L., Barbera G.A. (2016). The protein corona of circulating PEGylated liposomes. Biochim. Biophys. Acta-Biomembr..

[13] Benne N., Van Duijn J., Lozano Vigario F., Leboux R.J., Van Veelen T.P., Kuiper J., Jiskoot W., Slütter B. (2018). Anionic 1,2-distearoyl-sn-glycero-3-phosphoglycerol (DSPG) liposomes induce antigen-specific regulatory T cells and prevent atherosclerosis in mice. J. Control Release.

[14] Palchetti A.S., Pozzi D., Capriotti A.L., Barbera G.L., Chiozzi R.Z., Peruzzi G., Caracciolo G., Palchetti S., Pozzi D., Capriotti A.L. (2017). Influence of dynamic flow environment on nanoparticle-protein corona: From protein patterns to uptake in cancer cells. Colloids Surf. B Biointerfaces.

[15] Yasmin A., Ezeddine H., François D. (2023). The interplay between lysosome, protein corona and biological effects of cationic carbon dots: Role of surface charge titratability. Int. J. Pharm..

[16] Francesca L., Ilaria T., Alessandro M.D., Dominici F., Argentati C., Morena F., Torre L., Puglia D., Martino S. (2020). Novel Nanocomposite PLA Films with Lignin/Zinc Oxide Hybrids: Design, Characterization, Interaction with Mesenchymal Stem Cells. Nanomaterials.

[17] De L.M., Ferraro M.M., Hartmann R., Rivera P., Klingl A., Nazarenus M., Ramirez A., Parak W.J., Bucci C., Rinaldi R. (2015). Advances in Use of Capsule-Based Fluorescent Sensors for Measuring Acidification of Endocytic Compartments in Cells with Altered Expression of V-ATPase Subunit V1G1. ACS Appl. Mater. Interfaces.

[18] Ispanixtlahuatl-Meráz O., Delgado-Buenrostro N.L., Déciga-Alcaraz A., Del Pilar Ramos-Godinez M., Oliva-Rico D., López-Villegas E.O., Chirino Y.I. (2021). Differential response of immobile (pneumocytes) and mobile (monocytes) barriers against 2 types of metal oxide nanoparticles. Chem.-Biol. Interact..

[19] Zhu X., Sun J., Zhang Y., Sun X.L. (2016). Immunization with functionalized carbon nanotubes enhances the antibody response against mode antigen ovalbumin. Immunol. Lett..

[20] Takuma I., Safaa N., Megan V. (2024). Therapeutic strategies to target connective tissue growth factor in fibrotic lung diseases. Pharmacol. Ther..

[21] Chen D., Ganesh S., Wang W., Amiji M. (2019). The role of surface chemistry in serum protein corona-mediated cellular delivery and gene silencing with lipid nanoparticles. Nanoscale.

[22] Chen D., Parayath N., Ganesh S., Wang W., Amiji M. (2019). The role of apolipoprotein- and vitronectin-enriched protein corona on lipid nanoparticles for in vivo targeted delivery and transfection of oligonucleotides in murine tumor models. Nanoscale.

[23] Chatterjee S., Kon E., Sharma P., Dan P. (2024). Endosomal escape: A bottleneck for LNP-mediated therapeutics. Proc. Natl. Acad. Sci. USA.

[24] Carrillo C., Carril M., Parak W.J. (2017). Techniques for the experimental investigation of the protein corona. Curr. Opin. Biotechnol..

[25] Wilhelm S., Tavares A.J., Dai Q., Ohta S., Audet J., Dvorak H.F., Chan W.C. (2016). Analysis of nanoparticle delivery to tumours. Nat. Rev. Mater..

