# Correction: Wang et al. Impact of Protein Coronas on Lipid Nanoparticle Uptake and Endocytic Pathways in Cells. *Molecules* 2024, *29*, 4818

**DOI:** 10.3390/molecules31060930

**Published:** 2026-03-11

**Authors:** Rui Wang, Jing He, Yuhong Xu, Baowei Peng

**Affiliations:** 1 College of Pharmacy, Dali University, No. 2 Hongsheng Road, Dali 671003, China; 13611528427@163.com (R.W.); m15969529279@163.com (J.H.); 2 Yunnan Key Laboratory of Screening and Research on Anti-Pathogenic Plant Resources from Western Yunnan, Dali University, Xueren Road, Dali 671003, China

In the original publication [1], there was a mistake in Figures 1 and 2, as published. We have confirmed that, in the original manuscript, the nuclear image of the AAS sample in the first row of Figure 2 was incorrectly used. This image inadvertently duplicated the nuclear image of the 4 h sample shown in the second row of Figure 1. The correct Figure 1 and Figure 2 appear below. The authors state that the scientific conclusions are unaffected. This correction was approved by the Academic Editor. The original publication has also been updated.

## Figures and Tables

**Figure 1:**
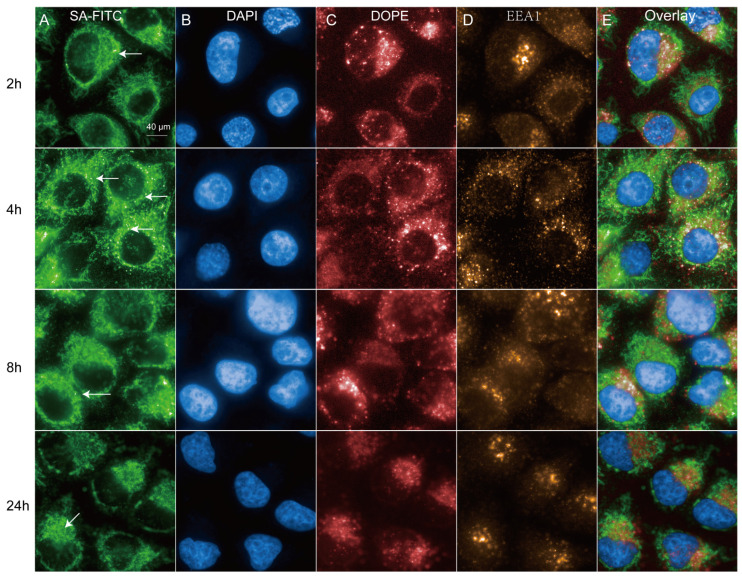
Characterization of intracellular uptake of dUTP-11-Biotin LNPs at different time points. (**A**) Endocytosis of LNPs DNA-FITC in HeLa cells pulsed with 2.5 µg DNA-FITC encapsulated in LNPs for 2 h, 4 h, 8 h, and 24 h. (**B**) HeLa cells were labeled with DAPI. (**C**) LNPs/lipids were labeled with DOPE-atto647. (**D**) LNPs/lipids were labeled with EEA1. Arrowheads point to LNP-DNA in perinuclear “cloud”, and arrows point to individual cytoplasmic endosomes. (**E**) Representative merged images (Overlay) at indicated time points.

**Figure 2:**
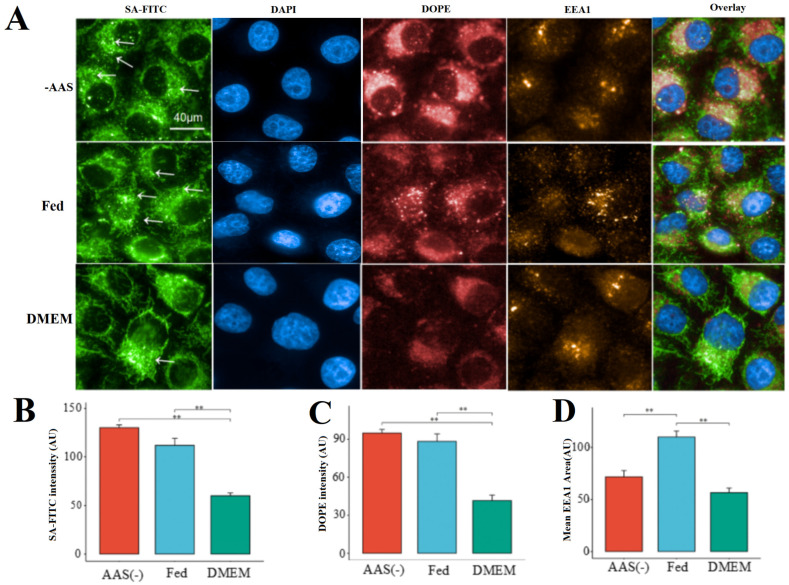
The internalization of LNPs varies depending on different nutrients. (**A**) Correlation of peripheral LNP endosomes with endocytosis activity in AAs(−) HeLa cells, Fed HeLa cells, and DMEM HeLa cells. (Arrows point to individual cytoplasmic endosomes.) (**B**) Quantification of SA-FITC intensity. (**C**) Quantification of DOPE intensity. (**D**) Quantification of EEA1 area. ** *p* < 0.01.
