# Supplementary figures and images for: Impact of Protein Coronas on Lipid Nanoparticle Uptake and Endocytic Pathways in Cells
Source: Molecules. 2024 Oct 11;29(20):4818. doi: 10.3390/molecules29204818 (PMC11510507; doi:10.3390/molecules29204818)

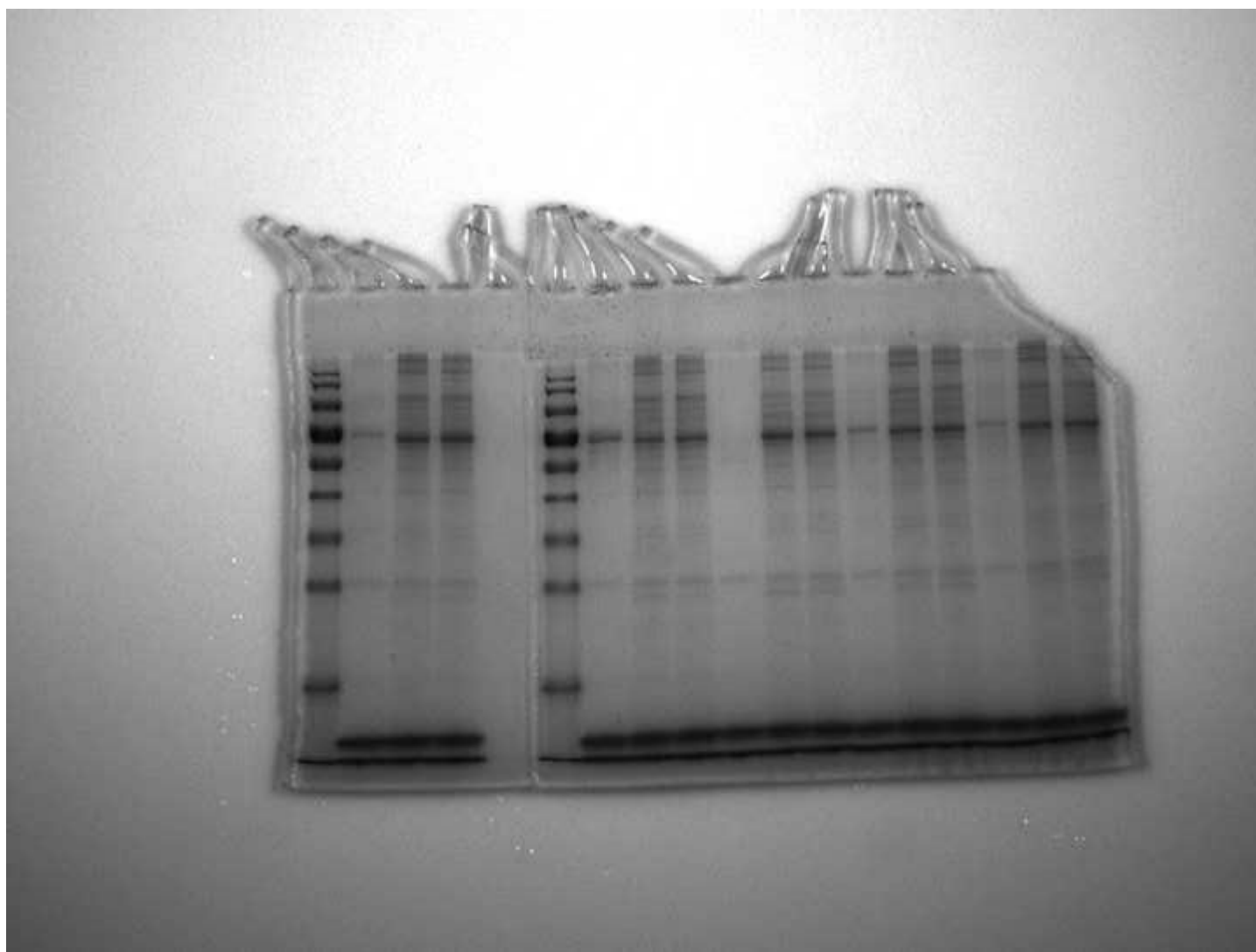

Supplement: Supplementary file 1 [file molecules-29-04818-s001.zip › Figure S1.pdf]
